# Composition and vertical distribution of organic matter in Central Indian Ocean sediment cores

**DOI:** 10.1038/s41598-023-49116-y

**Published:** 2024-01-25

**Authors:** Sruthi Thalayappil, Muhammed Nayeem Mullungal, Sajna Peediyakkathodi, Ratheesh Kumar C. S., Resmi Panikkaveettil, Salas P. M., Sujatha C. H.

**Affiliations:** 1 Department of Chemical Oceanography, School of Marine Sciences, Cochin University of Science and Technology, Kochi, Kerala 682016 India; 2 Environmental Sciences Program, Department of Biological and Environmental Sciences, College of Arts and Science, Qatar University, P.O. Box 2713, Doha, Qatar; 3 Kerala University of Fisheries and Ocean Studies, Panangad, Kerala India; 4 School of Environmental Studies, Cochin University of Science and Technology, Kochi, Kerala 682022 India

**Keywords:** Biogeochemistry, Environmental sciences, Ocean sciences

## Abstract

This study aimed to investigate the quality and quantity of organic matter (OM) using biochemical components (protein, carbohydrate and lipids) in the sediment cores collected from the Central Indian Ocean (CIOB) under the program Equatorial Indian Ocean Process Study Dynamics and Biogeochemistry (EIOPS). Total organic carbon (TOC) and biochemical parameters (carbohydrate, lipid, protein, Tannin and lignin) were determined in core I and core II, respectively. Total carbohydrates varied from 4.66 to 2557.32 mg/kg (average 459.31 mg/kg) and 142.23 to 821.56 mg/kg (average 380.01 mg/kg) in core I and II, respectively. In core I, PRT varied from 70.95 to 107.05 mg/kg, and the minimum and maximum content of CHO was 143.23 and 822.56 mg/kg. The maximum and minimum concentrations of BPC in core II were 786.32 and 381.07 at 0–10 cm depth, respectively, which corresponded to the concentrations of PRT, CHO, LPD, and Tannin. The results showed that PRT was statistically significant with the TOC and negatively correlated with the LPD and CHO, while LPD was highly significant with clay and silt grains. In most of the samples, the ratio of LPD to CHO ratio was > 1, which indicated higher productivity of benthic organisms inhabiting the CIOB.

## Introduction

The benthic region is vital to the marine environment since it represents the transition zone between the biosphere and the geosphere^1^. Particulate organic matter produced in the euphotic zone enters the water column, ultimately reaching the sea floor and fueling the benthic community. Also, bioturbation and biogeochemical cycling determine the fate of organic matter (OM) accumulated in sediments^2,3^. The quality of OM is essential from the perspective of both biogeochemical and trophodynamic, in which the former affect its burial rate, and the latter influences feeding strategies and an ecological link between pelagic and benthic ecosystem^4,5^. The quantity and quality of OM in sediments are of primary importance in determining the potentially available fraction to consumer organisms, ultimately influencing the food chain and benthic metabolism^6,7^.

Sedimentary organic matter (SOM) is composed of labile and refractory compounds, and its distribution and dynamics are controlled by complex biogeochemical processes, such as degradation, heterotrophic utilization, transformation, accumulation and export^8^. Refractory organic compounds have been characterized by lower degradation rates, which generally account for a significant portion of SOM and quickly accumulate in marine sediments^9^. Conversely, the labile fraction of OM consists of proteins, carbohydrates and lipids, rapidly mineralized and assumed to represent the fraction of OM more readily available to benthic consumers^10,11^.

The Indian Ocean receives more than half (2950 km^3^) of the runoff from the Bay of Bengal. Thermohaline alteration in the equatorial Indian Ocean (from 10° S to 10° N) is mainly due to surface circulation, precipitation and fresh water, and annual wind reversal patterns associated with the Asian monsoon system^12^. Southwest monsoon trigger upwelling along the coasts of Arabia, Somalia, Sri Lanka, and the southwest coast of India^13^, but in winter, the NE monsoon inhibits strong upwelling^14^. Strong seasonal winds during the southwest and northeast monsoon promote significant seasonal changes in hydrography and particle fluxes. The past variations in productivity are related to climate change through glacial/interglacial cycles and are linked to global parameters such as ice volume, deep-water circulation and continental climate. Glacial periods may experience less seasonality due to the weaker SW Indian monsoon and the more vigorous NE monsoon^14^, which may also reflect the productivity pattern in the Indian Ocean.

Recently^15^ studied the dynamics and bioavailability of phosphorous in the Central Indian Ocean (CIOB) region; however, the composition of OM and its dynamics are poorly understood in this region. The documentation of deep-sea OM dynamics will help understand the connection between nutrients and the ecosystem, bioavailability and impacts on marine biota. Hence, this study aimed to investigate the quality and quantity of OM using biochemical components (protein, carbohydrate and lipids) in the sediment cores collected from CIOB.

## Materials and methods

### Study area

The investigation was undertaken in the CIOB under the program “Equatorial Indian Ocean Process-Study Dynamics and Biogeochemistry (EIOPS). The Central Indian Ocean Basin (CIOB) has an area of 5.7 × 10^6^ km^2^ and is bordered by the 90°E Ridge in the east, the Mid-Indian Ridge in the west and the South-western Ridge in the south. The samples were collected on board by O.R.V. Sagar Kanya on Equatorial Indian Ocean Cruise (SK-267) from January to February 2010.

**Figure 1:**
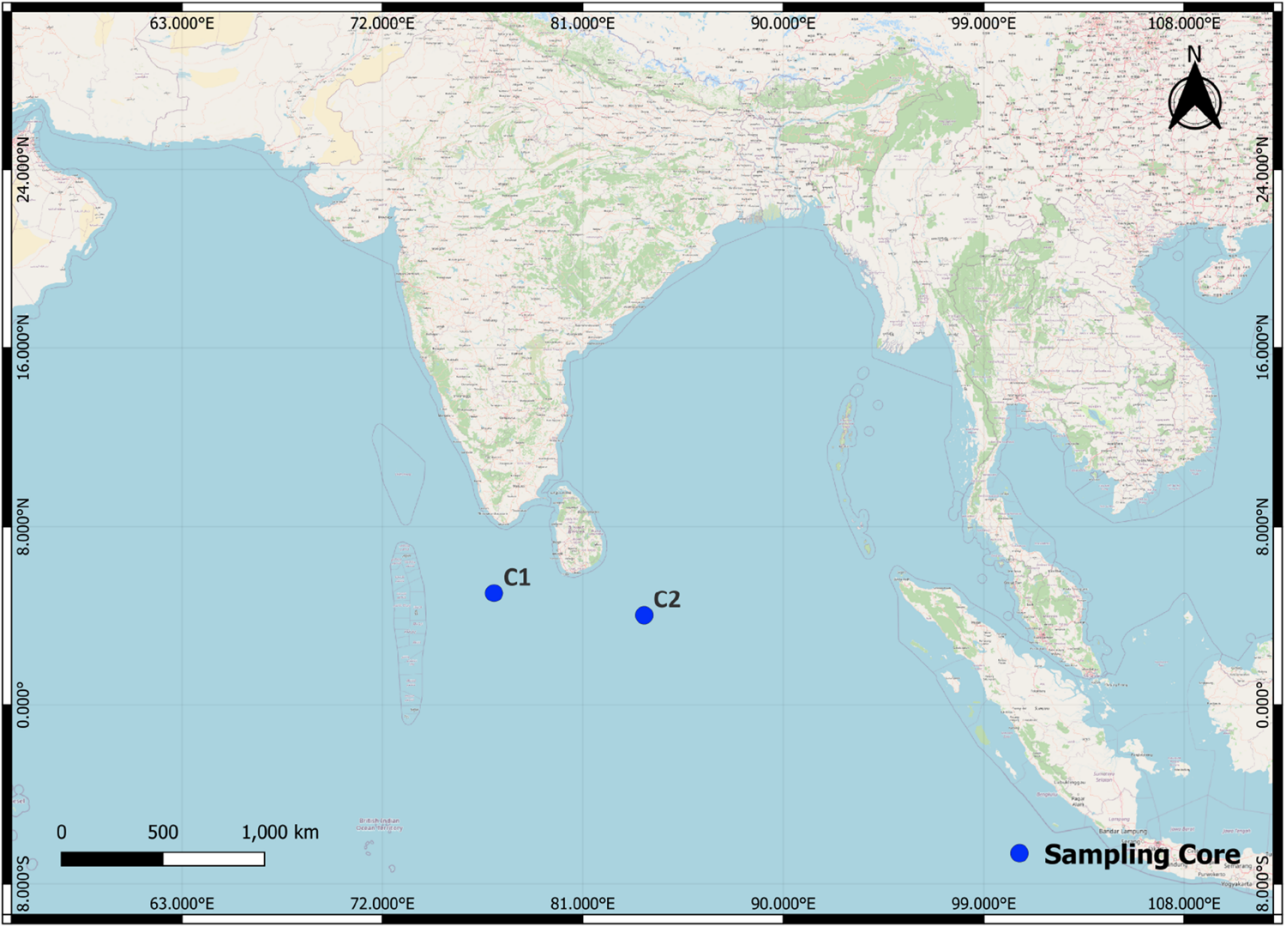
Study area; core I (C1) and core II (C2), The Map created using the Free and Open Source QGIS” by using Version 3.32.3 (https://www.qgis.org/en/site/).

Sediment core samples were collected from two different regions (Fig. 1); core I was 565 cm long, while core II was only 30 cm. Core I was collected from the CIOB, dominated by terrigenous sediments derived from the northwest part of the basin. Core II samples were collected from the eastern side of the Afanasiy-Nikitin Seamount (ANS), located at 3° 2′ 2.1" S latitude and 83° 5′ 19.2" E in the Equatorial East Indian Ocean^16^. Core II was obtained from the southern region of the CIOB, known for its significant presence of manganese nodules and Ferromanganese crusts^17^, which posed constraints on the core's sampling depth.

### Sampling and analysis

Gravity corer (10 cm internal diameter and 10 m long) used for the collection of sediment samples. Core I was sectioned into 57 subsamples, while core II was sectioned only into three subsamples (Fig. 1). The undisturbed middle portion of each subsample was transferred to wide-mouthed polythene bottles and taken to the laboratory for further analysis under ice-cold storage conditions. Furthermore, samples were kept in a deep freezer at − 20 °C prior to analyses. Each subsection of the core was freeze-dried in a flow type of Freeze drier (Beetta Instruments and Equipment co., India). The dried sediment samples were homogenized and sieved through a mesh-size screen of 63 μm. These sediment samples were subjected to further analysis of total organic carbon (TOC) and biochemical parameters (Tannin and lignin, protein, carbohydrate and lipid). The Sum of concentrations of total protein (PRT), total carbohydrates (CHO), and total lipids (LPD) together constitute the labile organic matter (LOM).

The percentage composition of sand, silt, and clay was determined by wet sieving^18^. TOC level was assessed by CHNS Analyzer (Vario EL III CHNS Analyzer, Thermo Spectronic) after treating with 10% HCl to remove inorganic carbon and repeated two/three times in order to ensure the complete exclusion of carbonates^19^. The redox potential of the sediment was measured by the potentiometric method^20^. Spectrophotometric methods were employed for the determination of CHO, PRT and LPD. The concentration of CHO in sediments was estimated by the phenol sulphuric acid method^21^, using glucose as the standard. Protein analyses were carried out^22,23^ to account for the reactivity of phenolic compounds and use bovine albumin as the standard. Estimation of total lipid was done by the sulphovanillin method^24^. Hydroxylated aromatic compounds-Tannin and lignin (T + L) were estimated by the method detailed in^25^ and modified by^26^. Protein, carbohydrate and lipid concentrations were converted to carbon equivalents using the following conversion factors: 0.49, 0.40 and 0.75 g of C∕g, respectively^27^. The sum of protein, carbohydrate, and lipid carbon was referred to as biopolymeric carbon (BPC)^10,28^ . All the containers involved in the chemical analysis were cleaned with a nutrient-free detergent, and the cleaning solution was prepared by dissolving 36 g of ammonium peroxydisulfate (NH_4_)S_2_O_2_ in a loosely stoppered 2.2 L bottle of 98 wt% sulphuric acid^29^. Containers used in the sampling of sediments (after slicing the core) were twice with the Milli-Q water before sample collection. Samples were quantified in triplets to ensure the reproducibility of the data. Measurement/ estimation of all the variables was carried out in triplicate, and the average value/concentration was reported.

The vertical distribution graph was plotted using Origin Pro. Using Origin Pro, Pearson correlation and Principal Component Analysis (PCA) were carried out to assess the relationship between OM components with sediment grain size and total organic carbon (TOC). Pearson correlation analysis was performed to test for possible relationships among the investigated variables.

## Results

**Figure 2:**
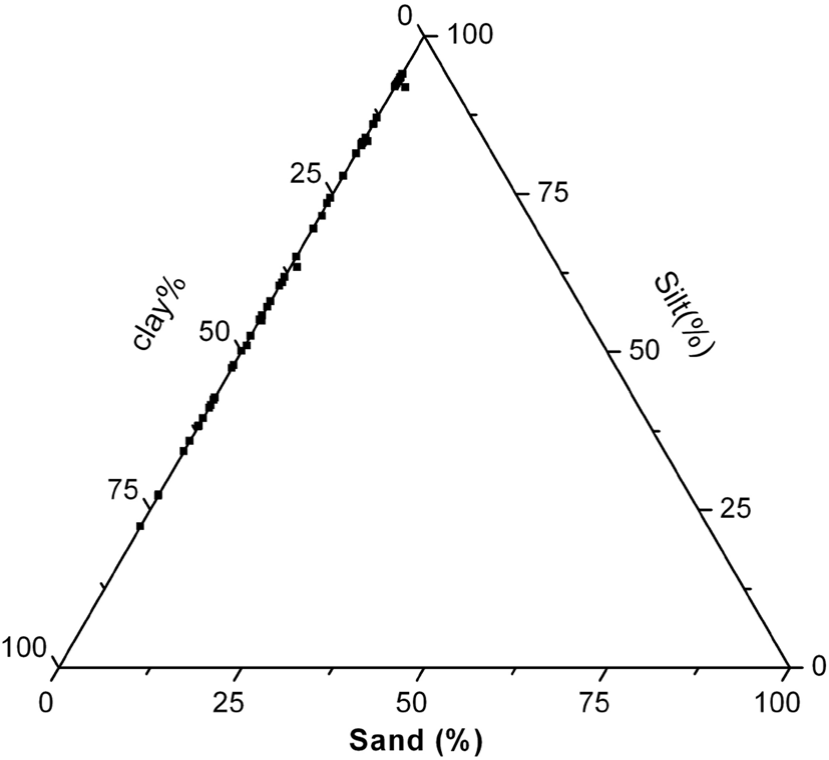
Trilinear diagram showing grain size distribution in Core I.

**Figure 3:**
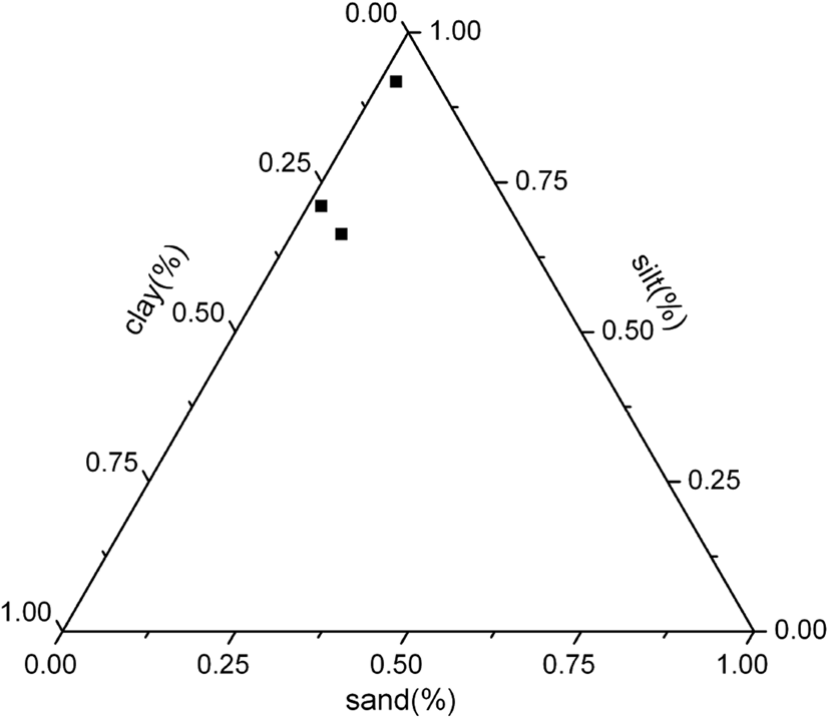
Trilinear diagram showing the grain size distribution of core II.

Texture analysis revealed the dominance of finer particles along the core, shown in the trilinear diagram (Figs. 2 and 3). Considerable assortments of textural grades were observed in the sediment. The data points in the trilinear plot gradually change from silt to silty clay towards the bottom of the core.

The grain size of core I varied from 0 to 1.48% for sand, 22.36 to 93.96% for silt and 5.94 to 77.63% for clay. Even though a high percentage of fine fractions were exhibited throughout the material, the core exhibited two different textural facies. The top horizon (0 to 420 cm) in the core I exhibited a higher silty horizon, whereas the bottom horizon (430–565 cm) dominates in clayey facies.

The core II collected in the eastern part of the basin exhibited dominance of single facies of fine fraction grains, i.e. silt. The top layer (0–10 cm) of core II was rich in silt (more than 90%), and textural facies in the bottom layers (20–30 cm) were of clayey silts. The sand fraction was comparatively higher than the core I sampled and varied from 1.89 to 7.15%. Silt and clay fractions varied from 66.35 to 91.77% and 5.84 to 27.08%, respectively.

**Figure 4:**
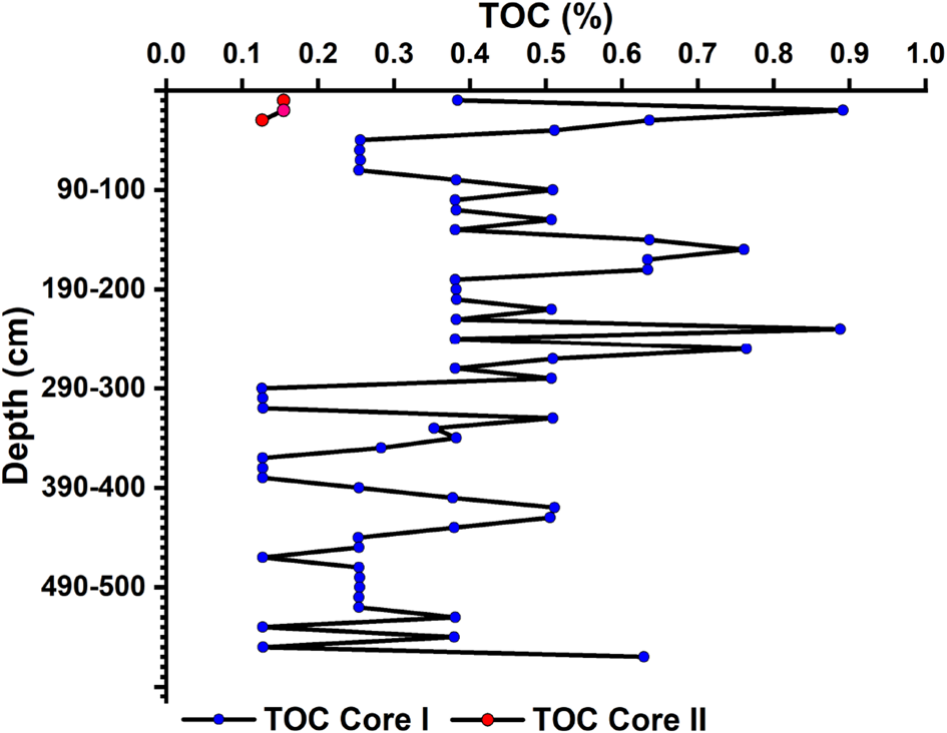
Variations of total organic carbon in core I and core II.

Information on (TOC) content in sediments is vital to assess the organic fraction's role in the transport, deposition, and retention of metals and nutrients. The distribution pattern of TOC in both the cores with depth is depicted in Fig. 4. In core I, the maximum TOC content was 0.89%, noted at a depth interval of 10–20 cm. The minimum was 0.12% observed at a depth interval of 290–300 cm, slightly higher than the global average (0.2%) of deep-sea sediments^30^. The observed range for redox potential was noted to be from − 341 (440–450 cm) to − 18 mV (170–180 cm), providing clues for the degradation of OM in sediments.

TOC content in core II ranged from 0.15 to 0.12% at a depth interval of 10–20 cm and 20–30 cm, respectively. TOC content was pronounced in silt and clay-dominated sediments with lower E^h^. Higher TOC content in fine-textured sediments than the coarse-textured can be trumpeted that differences in the carbon sink, rather than long-term decomposition dynamics. In an aquatic environment, the content of TOC increases as the texture becomes more delicate. Clay particles act as a shield to rapidly decomposing compounds from microbial activity through processes of encrustation and entrapment^31^.

**Figure 5:**
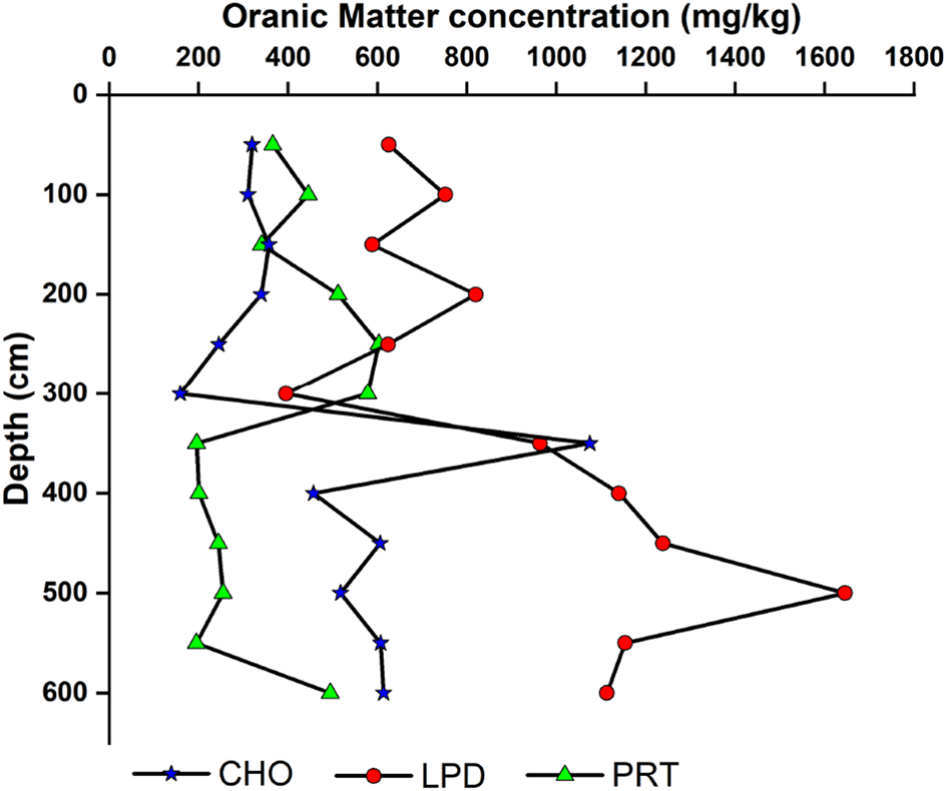
Down core variation in Organic Matter (CHO, LPD, PRT) in core I.

Tables 1 and 2 show the vertical distribution of PRT, CHO, LPD, Tannin & lignin, and BPC. Among the biochemical constituents (as shown in Tables 1 and 2), the total carbohydrate content recorded from 4.66 to 2557.32 mg∕kg in core I and from 142.23 to 821.56 mg∕kg in core II (Fig. 5). Notably, a significant variation was observed at a depth of 350 cm (1074.7 mg/kg) in core I. In addition, the concentration of CHO is higher in the surface sediment of core II.

**Table 1:** Vertical distribution of organic matter in core I (mg/kg).

Depth (cm)	CHO	PRT	LPD	Tan and lignin	BPC
0–10	58.34 ± 2.31	235.3 ± 1.68	465.2 ± 5.45	9.98 ± 0.32	768.82 ± 6.31
10–20	112 ± 3.11	219.1 ± 2.94	614.2 ± 5.77	2.55 ± 0.05	947.85 ± 8.63
20–30	1084.94 ± 24.75	408.35 ± 3.98	609.7 ± 5.16	3.48 ± 0.11	2106.47 ± 24.94
30–40	195.6 ± 3.22	434.45 ± 4.65	601.2 ± 3.94	7.66 ± 1.19	1238.91 ± 16.81
40–50	144.64 ± 2.48	531.5 ± 4.99	835.6 ± 7.64	5.56 ± 1.03	1517.31 ± 18.34
50–60	410.64 ± 4.57	528 ± 9.00	663.9 ± 15.37	8.11 ± 1.09	1610.66 ± 21.56
60–70	401 ± 5.01	1031 ± 14.31	221.3 ± 1.98	3.71 ± 0. 48	1657.01 ± 20.37
70–80	202.98 ± 2.98	403.35 ± 3.54	1332 ± 23.48	1.15 ± 0.35	1939.49 ± 63.80
80–90	515.64 ± 6.34	183 ± 2.55	591.7 ± 7.14	3.93 ± 0. 76	1294.27 ± 45.19
90–100	21.02 ± 0.95	78.45 ± 1.64	948.5 ± 28.53	4.38 ± 0.87	1052.35 ± 21.11
100–110	49.02 ± 1.02	155.6 ± 4.03	772.3 ± 18.87	0	976.92 ± 12.20
110–120	21.02 ± 0.95	509 ± 13.25	682 ± 14.83	0	1212.02 ± 46.40
120–130	601.96 ± 7.21	661 ± 14.32	754.3 ± 9.73	0	2017.26 ± 57.97
130–140	240.32 ± 2.55	209.15 ± 4.87	433.6 ± 12.81	0	883.07 ± 10.72
140–150	872.64 ± 7.94	169.3 ± 2.57	298.1 ± 9.41	5.79 ± 1.08	1345.83 ± 42.14
150–160	237.98 ± 1.32	64.75 ± 3.73	722.6 ± 12.07	0	1025.33 ± 16.06
160–170	1182.94 ± 28.65	668.5 ± 7.79	975.6 ± 14.39	0	2827.04 ± 50.33
170–180	146.98 ± 6.22	981 ± 21.59	1247 ± 23.75	0	2374.98 ± 74.66
180–190	18.66 ± 1.50	480.55 ± 1.28	49.68 ± 3.44	0	548.89 ± 09.84
190–200	112 ± 3.84	364.75 ± 8.48	1102 ± 16.77	4.16 ± 0.98	1582.91 ± 40.08
200–210	338.32 ± 4.33	618.5 ± 10.81	131 ± 9.99	0	1087.82 ± 33.21
210–220	226.32 ± 2.15	207.9 ± 9.87	222 ± 5.61	0	656.22 ± 20.80
220–230	326.64 ± 3.05	140.65 ± 3.17	483.3 ± 8.73	0	950.59 ± 27.84
230–240	189.02 ± 2.61	1268.5 ± 47.48	1260 ± 27.19	0	2717.52 ± 64.29
240–250	144.64 ± 3.54	778 ± 19.09	1020 ± 24.08	1.86 ± 0.36	1944.5 ± 60.75
250–260	34.998 ± 1.33	169.3 ± 3.67	85.81 ± 6.14	0	290.12 ± 8.13
260–270	189.02 ± 6.05	698.5 ± 25.75	284.5 ± 10.11	0	1172.02 ± 30.59
270–280	72.32 ± 3.33	952.5 ± 24.21	799.4 ± 9.78	0	1824.22 ± 56.69
280–290	394.32 ± 2.89	783 ± 18.67	573.6 ± 6.19	0	1750.92 ± 59.77
290–300	102.66 ± 3.02	286.35 ± 6.07	234.9 ± 3.18	4.18 ± 0.85	628.09 ± 23.84
300–310	328.98 ± 4.66	297.55 ± 4.37	867.2 ± 29.79	0	1493.73 ± 58.09
310–320	4.66 ± 0.35	206.65 ± 6.67	1224 ± 32.66	0	1435.31 ± 15.86
320–330	1455.92 ± 56.43	216.62 ± 4.31	578.1 ± 16.81	0	2250.64 ± 61.51
330–340	2557.32 ± 76.32	231.5 ± 5.09	519.4 ± 9.47	0	3308.22 ± 74.19
340–350	1026.62 ± 53.02	23.65 ± 3.69	1630 ± 21.48	8.11 ± 1.03	2688.39 ± 65.11
350–360	758.3 ± 8.69	336.1 ± 5.37	1700 ± 26.94	0	2794.4 ± 68.43
360–370	433.98 ± 5.34	220.35 ± 10.22	1060 ± 20.11	0	1714.33 ± 30.06
370–380	321.1 ± 6.31	11.20 ± 1.59	1280 ± 32.28	0	1612.30 ± 30.45
380–390	202.98 ± 7.94	338.6 ± 11.84	1420 ± 27.74	0	1961.58 ± 39.56
390–400	566.98 ± 10.33	97.1 ± 5.04	240 ± 5.11	2.31 ± 0.19	906.39 ± 19.32
400–410	863.3 ± 13.08	246.5 ± 16.71	1470 ± 38.08	0	2579.8 ± 100.12
410–420	102.66 ± 5.33	44.815 ± 94.89	1320 ± 26.81	0	1467.47 ± 50.02
420–430	426.98 ± 6.37	262.7 ± 10.03	1098 ± 28.55	0	1787.68 ± 44.05
430–440	1049.94 ± 73.06	371 ± 4.00	844.6 ± 17.12	0	2265.54 ± 80.09
440–450	585.64 ± 8.46	296.3 ± 3.08	1459 ± 34.94	1.63 ± 0.33	2342.57 ± 87.85
450–460	207.66 ± 6.98	242.75 ± 3.84	1440 ± 38.19	0	1890.41 ± 44.10
460–470	622.96 ± 9.66	465.6 ± 5.16	1427 ± 32.26	0	2515.56 ± 67.10
470–480	366.32 ± 3.78	244 ± 13.10	1283 ± 29.99	0	1893.32 ± 62.78
480–490	685.96 ± 4.32	66 ± 5. 58	1892 ± 42.74	0	2643.96 ± 96.89
490–500	702.3 ± 6.20	253.95 ± 6.01	2186 ± 46.45	4.84 ± 0.57	3147.09 ± 102.34
500–510	713.96 ± 7.66	174.3 ± 7.63	853.6 ± 21.64	0	1741.86 ± 77.44
510–520	797.96 ± 6.35	156.85 ± 10.10	1373 ± 20.45	0	2327.81 ± 98.56
520–530	440.98 ± 4.36	287.55 ± 12.09	1418 ± 36.57	0	2146.53 ± 54.93
530–540	277.66 ± 5.31	93.35 ± 2.53	1536 ± 40.50	0	1907.01 ± 44.62
540–550	804 ± 14.33	262.7 ± 3.19	587.1 ± 8.31	1.38 ± 0.27	1655.19 ± 53.04
550–560	669.64 ± 7.67	400.85 ± 5.98	867.2 ± 16.88	0	1937.69 ± 66.32
560–570	557.64 ± 6.22	587.5 ± 6.18	1359 ± 22.92	0	2504.14 ± 74.19

**Table 2:** Vertical distribution of organic matter in core II (mg/kg).

Depth (cm)	CHO	PRT	LPD	Tan and lignin	BPC
0–10	821.56 ± 14.03	107.05 ± 5.04	0	17.13 ± 1.32	928.61 ± 5.22
10–20	142.23 ± 2.10	89.65 ± 6.34	914 ± 19.77	1.62 ± 0.52	1145.88 ± 13.55
20–30	176.24 ± 8.77	70.95 ± 2.14	595 ± 12.43	3.71 ± 0.33	842.19 ± 22.71

**Figure 6:**
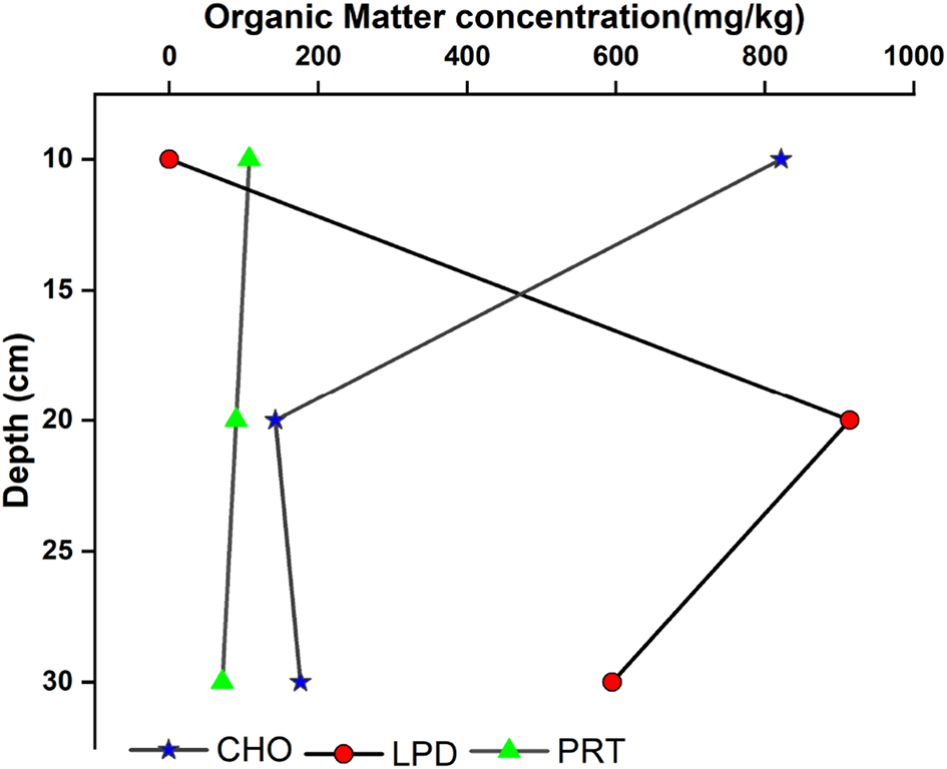
Down core variation in Organic Matter (CHO, LPD, PRT) in core II.

The PRT content in core I ranged between 11.20 and 1268.5 mg/kg for the samples from 370 to 380 cm and 230 to 240 cm depths, respectively. PRT followed an asymmetrical zig-zag pattern and showing evenness in between 350 and 550 cm. In core II **(**Fig. 6), the Concentration of PRT varies from 70.95 to 107.05 mg/kg (average 89.22) follows a straight line.

The surface sediment layer was devoid of LPD, and a maximum of 595 mg/kg was found at 30 cm depth. Markedly highest concentration at 20 cm of depth (914 mg/kg). The LPD has irregular zig-zag pattern; in which increase of LPD concentration from 350 to 500 cm and then decrease.

**Figure 7:**
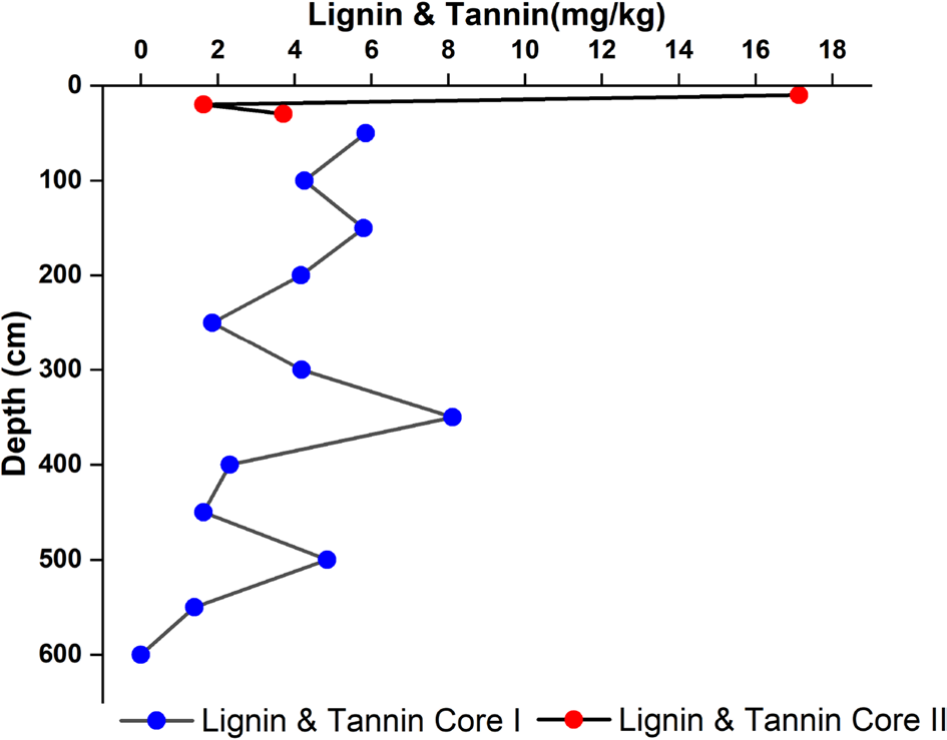
Down core variations of Tannin and lignin in core I and core II.

Lignin and tannin concentration (Fig. 7) in core I was 1.15–9.98 mg/kg (average 4.46 mg/kg) showing zig-zag trend with highest content at the surface sediment. Concentration in core II was 1.62–17.13 mg/kg (average 7.49 mg/kg); considerable amount at the surface.

**Figure 8:**
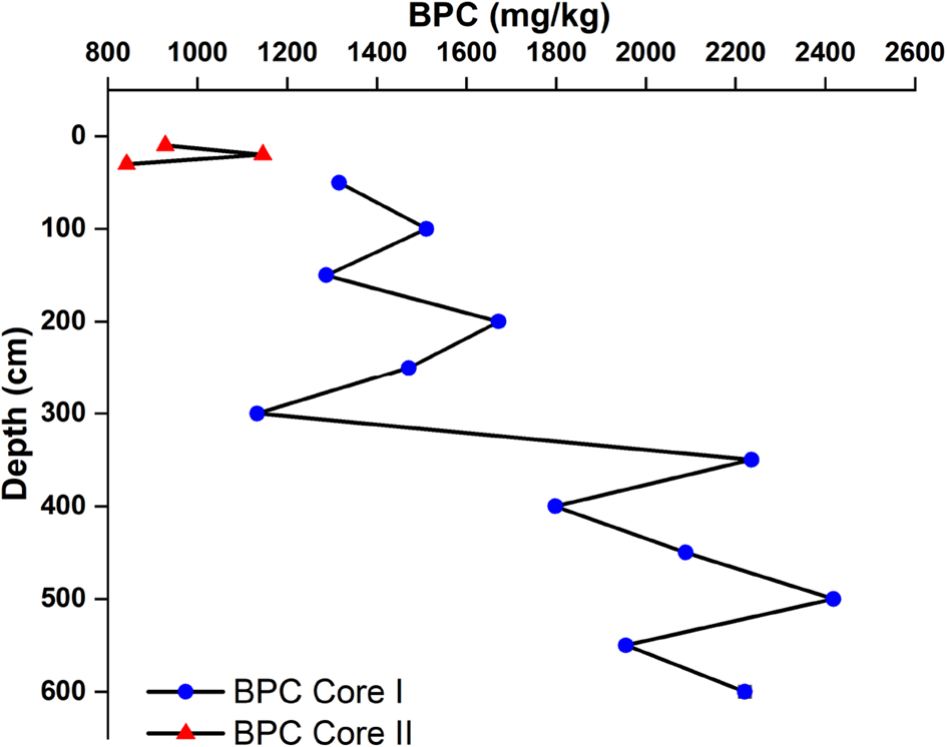
Vertical distribution of BPC in core I and core II.

The percentage of BPC (Fig. 8) was found to be low at 250–260 cm depth (0.02%). Core I observed the maximum BPC value at 490–500 cm depth (0.20%). The maximum and minimum percentage of BPC in core II were found as 1145.88 and 842.19 mg/kg, respectively. The contribution of BPC to TOC was lower at bottom layers, and then it showed a zig-zag trend towards the bottom of the core.

**Figure 9:**
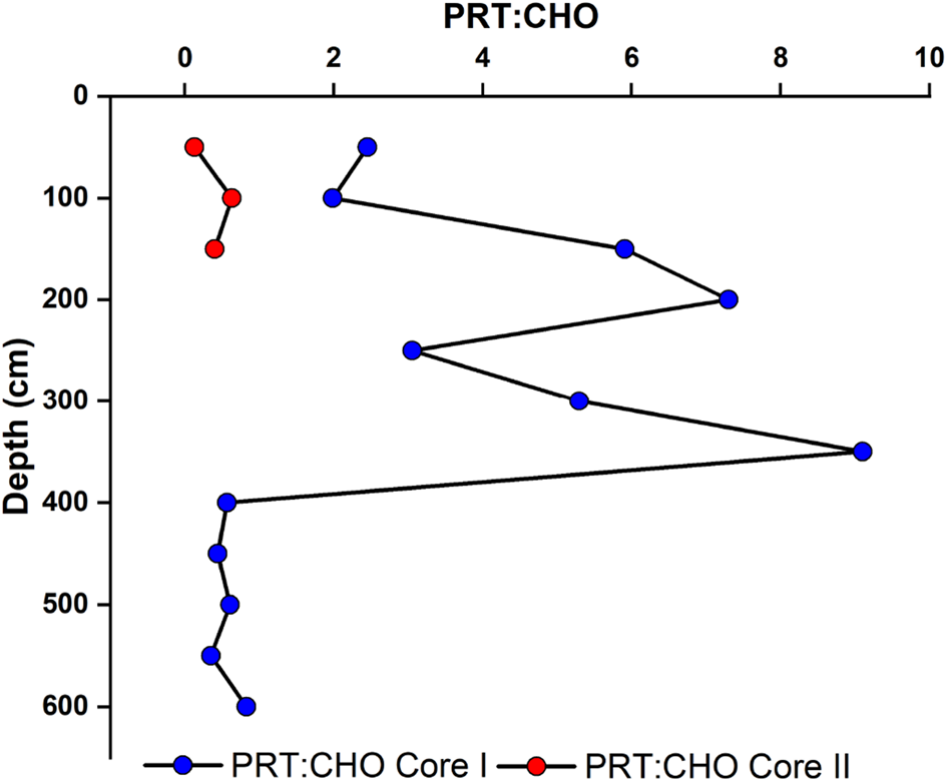
Vertical distribution of PRT: CHO in core I and core II.

**Figure 10:**
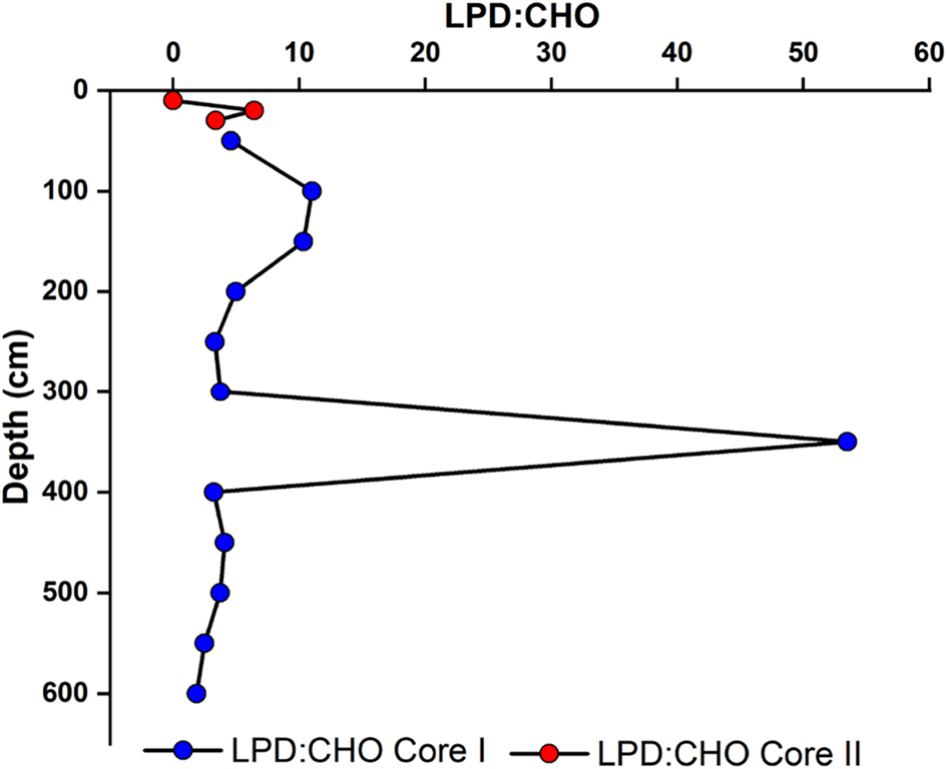
Vertical distribution of LPD: CHO in core I and core II.

The protein to carbohydrate ratio (PRT: CHO) showed a maximum value of 44.35 at 310–320 cm depth and a minimum value of 0.02 at 340–350 cm depth in core I. Nevertheless, this PRT: CHO ratio in core II varied from 0.13 to 0.63. In core I, the highest LPD: CHO ratio is found at 310–320 cm depth, and the value is 262.66, whereas the minimum value of 0.20 was found at a depth of 330–340 cm. In core II, the highest value, 6.43, was found at 20 cm depth and was negligible at 0–10 cm depth (Figs. 9, 10).

### Statistical analysis

**Figure 11:**
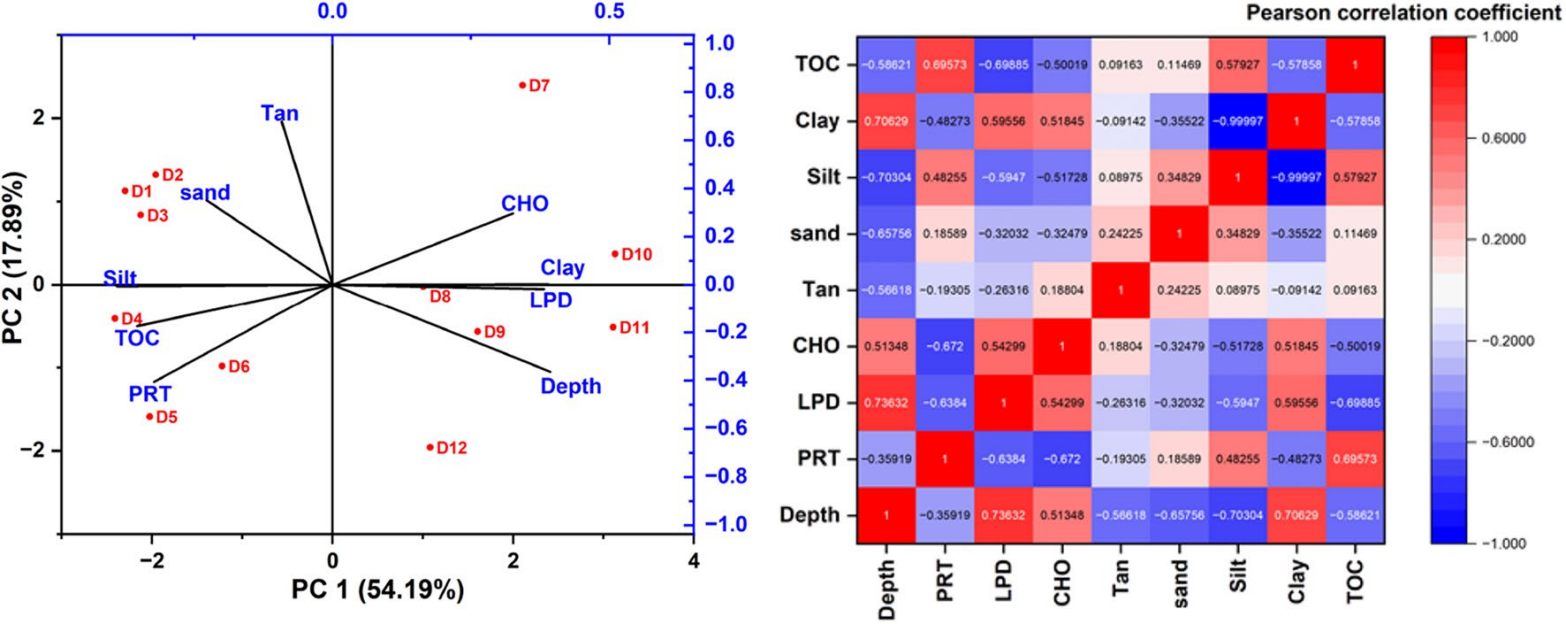
PCA and Pearson correlation matrix (Heat map) for Core I (Depth represented as D).

**Figure 12:**
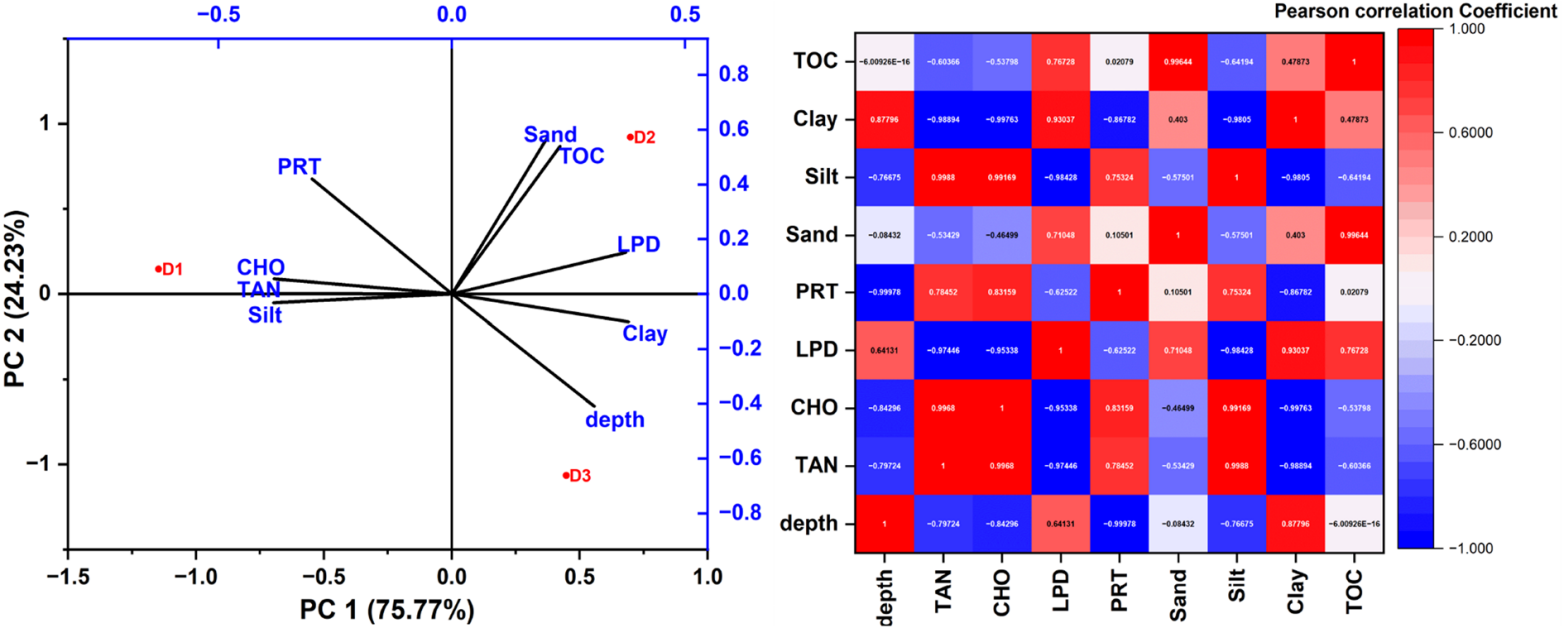
PCA and Pearson correlation matrix (Heat map) for Core II.

Principal component analysis (PCA) was performed to identify and interpret the relation among biochemical compounds, grain size TOC and depth. Pearson correlation was plotted as a heat map (Figs. 11 and 12). Statistical analysis of core I explained that the total variance was (PC1 and PC2) 72.08%, with PC1 accounting for 54.19% and PC2 accounting for 17.89% of the total variance. According to the Pearson correlation, in core I (Fig. 11), PRT was statistically significant with the TOC (r = 0.695, *p* = 0.011)^32^ and negatively correlated with the LPD (r =  − 0.638, *p* = 0.025) and CHO (r =  − 0.672, *p* = 0.016). LPD is hardly increasing with increasing depth (r = 0.736, *p* = 0.006). LPD is highly significant with the clay (r = 0.595, *p* = 0.041)^33^ and negatively correlated with the silt (r =  − 0.594, *p* = 0.041) and TOC (r =  − 0.698, *p* = 0.011). Tannin and lignin decrease with an increase in depth (r =  − 0.566, *p* = 0.054).

The total variance of Core II, explained as PC1 and PC2 (Fig. 12), was 100%, with PC1 accounting for 75.77% and PC2 accounting for 24.23% of the total variance. In the case of core II, CHO is highly correlated with the clay (r =  − 0.99 *p* = 0.043)^32^. Tannin and lignin correlated with the silt grain ((r = 0.998, *p* = 0.031) and PRT is correlated with the depth (r =  − 0.99 *p* = 0.013).

The strong relationships between texture and TOC with PRT, CHO, LPD and Tannin were pointed towards the adsorption and diagenetic process controlling the distribution of the biochemical components in the sedimentary environment. Interrelationships among the biochemical component each other point towards a common source and similar behaviour in the marine environment.

## Discussion

CIOB was a less oxidizing environment with a higher clay supply and high organic carbon content. The abyssal plains of CIOB were developed by turbidity currents, which disperse the terrigenous sediments from the Ganges–Brahmaputra system as far south as 8°S. It is mainly responsible for the variation in the depositional flux of clay^17,34^. More oxygenation, high productivity and less sedimentation rate were noticed in the southern region of CIOB^35^. The monsoon climate in the Himalayan region results in the very rapid transport of sediment to the Indian Ocean^36^. The productivity changes are related mainly to the Indian monsoon system, upwelling and oxygenation changes. Core I was dominated by terrigenous sediments received from the northwest part of the basin consisting of ~ 90% of the clay minerals, which are probably derived from the Himalayan Rivers through the Bay of Bengal. Core II was collected from the eastern part of the basin with abundant nodules, i.e., from the eastern side of the Afanasiy-Nikitin Seamount.

Textural characteristics in core I showed lower sand content (Fig. 2). The sand fraction was greater than 1% in only one sample (90–100 cm). The silt was the dominant fraction at the top of the core, and the variation of silt showed a zigzag pattern. It is likely that such a shift in sediment grain size and OM content could be due to larger proportions of terrigenous material in the intermediate deposits. The variation was mainly recorded in the finer fraction, i.e., clay and silt. Low clay flux in the Bay of Bengal could be associated with glacial climatic regimes and changes in the Indian summer monsoon. From 90 to 240 cm, variation in texture was almost constant. The change in texture was also noticed at 240 cm^37^ reported that slowly accumulating mud turbidites dominated between 7 to 0.9 Ma and were switching over to silty turbidites younger than 0.9 Ma. Sudden change of TOC occurred at 240 cm to 350 cm, which may have been caused by variations in sea level, changes in the depocentres between the proximal and distal basins, internal lobe switching, etc^36,38,39^. The proportion of silt was found to be decreased in the 240–350 cm layer. 350–420 cm section of the core (exception 360–370 cm) again showed variation in texture, with a higher % of silt as compared to the above section. Clay content increased to 500–510 cm and decreased to the bottom of the core. In core II, the sand fraction was slightly higher than in core 1 (< 8% only). The proportion of silt was found to be high in the surface sample, then decreased slightly (< 71%) down the core. The biochemical composition of the SOM could be assumed as an estimate of the material potentially available to benthic consumers^28,40,41^.

OM can be used qualitatively to assess the nature of the depositional area, which reveals the changes in past productivity^42,43^. The processes controlling the preservation and burial of OM are mainly assumed to be primary productivity, vertical fluxes, sedimentation, chemical stability, and degradation rates of particulate organic carbon and bottom water oxygen concentrations^44–46^. Higher TOC was noticed towards the mid of core I, and the down core variation recorded a zigzag pattern. The low productivity at the surface sediments may result either from weakening the NE monsoon or from the onset of the SW monsoon^47^. The sedimentation rate of the study region (= 3 cm/year) has already been reported^48^. Higher solar radiation at 9000 years BP leads to a more vigorous Asian summer monsoon circulation, which resulted in higher upwelling in the Arabian Sea. TOC content decreases due to the weakening of the upwelling process linked to the SW monsoon, which is reinforced at the beginning of the Holocene^49^. Reduction in southwest monsoon was reported during ≈15.7 ky, which may have resulted in the low TOC in the deglaciation period^50^. The prominent peak in TOC was noticed at 140–150 cm to 170–180 cm. An erosional event in the Himalayas was reported around 0.5 Ma, which may enhance TOC in the study region^51^. OM in the deep layers could be linked to sea surface circulation and NE winter monsoon, considered the major feature during the last glacial time.

At 230–240 cm, the TOC value recorded the maximum, which indicated a major event happened at that stage. The layer 250–260 cm also recorded elevated TOC, which resulted from a massive erosional event from the Himalayas caused by the tectonic activity during ≈0.8 Ma^52^. From 250 to 560 cm, the down core variation in TOC displayed a decreasing trend, then increased and followed a zigzag pattern. Decrease in TOC for post − 0.9 Ma was reported due to a lower abundance of C4 plants in the Ganga–Brahmaputra basin^53^. Another mass landslide in the Himalayan mountain range ≈, 1.7 Ma, may contribute to higher TOC^54^ in CIOB at 560–570 cm. In core II, TOC was very low due to the geographical features of the region (eastward region of the central Indian Ocean), which may not receive terrestrial influx from the Ganga–Brahmaputra river basins. In core II, a favourable condition for nodule development can be inferred.

The average concentration of PRT and LPD were high at an interval of 50–80 cm than at 0–30 cm. The sediment layer 50–80 cm represents the LGM period, which recorded elevated average concentration for lipid (573.6 mg/kg) and protein content (696.83 mg/kg) than Holocene (563.03 mg/kg and 287.58 mg/kg for lipid and protein content respectively) period (0–30 cm). High PRT levels can be linked to primary productivity^11^ or bacterial activity. The depth interval of 300 to 570 cm of the core I was recorded with lower protein content, but the lipid content was higher in this region. This lower protein content in the region indicated their utilization by heterotrophic microbes^55,56^. The higher LPD content may reflect in situ productivity as well as diatoms and faecal pellets of zooplankton are assumed to be important carriers of lipids to marine sediments^57^.

The presence of carbohydrates is ubiquitous and can be present > 75 wt% in vascular plants, > 40 wt% of bacteria^58^ and 20–40 wt% in planktons^59^. The top of the core recorded an elevated average concentration of carbohydrates (418.42 mg/kg and 318.76 mg/kg during the Holocene and LGM period, respectively). The concentration of carbohydrates was found to be higher at a depth interval of 300–570 cm. The concentration of PRT exhibited its maximum at 330–340 cm due to better preservation. The concentration of biochemical components was almost consistent at 200–300 cm. Lower carbohydrate levels were observed compared to protein and lipid throughout core I. In conditions that favour OM accumulation, i.e. high primary production and reducing conditions, efficient sugar consumption by the microbiota prevents the carbohydrates from contributing significantly to the preserved OM. However, these polymers, present in relatively low amounts in sediments, are a useful tool for studying biological and physicochemical processes in the aquatic environment.

The protein-to-carbohydrate ratio (PRT: CHO) has been used as an index to determine the origin of material present in sediments and to determine the age of SOM^40,60^. The samples from the top of the core up to 330 cm revealed a zig-zag pattern (Fig. 9) for PRT to CHO ratio, with the highest value at 310–320 cm. In the core, a higher PRT to CHO ratio could be due to better preservation of proteins. In addition, poor mineralized material tends to be colonized by bacteria rich in proteins^61^, which would increase the protein content of these materials and the index value.

An erosional event in the Himalayas was reported around 0.5 Ma, which contributed TOC^50^, and organic materials to the CIOB, which may lead to higher bacterial activity. Degradation of organic material at the water–sediment interface may lead to low preservation of OM in the sediment core^62^. However, the protein concentration was high, which may be due to higher bacterial activity in the sediment layer and hence showed an elevated PRT to CHO ratio. The increase in the nutritive value of the sediments is also indicated by the increase in the protein/carbohydrate ratio. The ratio ranges between < 0.1 in oligotrophic deep-sea sediments (500–2400 m depth) in the Eastern Mediterranean Sea) to higher than 10 in coastal Antarctic sediments^63^**.** It was found to be higher than the Antarctic sediment in some of the subsamples.

LPD content and the LPD/CHO ratio have been used as good indexes to describe the energetic (or food) quality of the organic contents in the sediment^64,65^. Furthermore, LPD concentration has been associated with the most labile fraction of sedimentary organics, and it was considered the best descriptor for meiofauna abundance and biomass over enzymatically hydrolyzable amino acids (EHAA) or PRT contents^66,67^. In most samples, LPD to CHO ratio was > 1, indicating higher productivity of benthic organisms inhabiting the CIOB. The sample 310–320 cm showed the highest LPD to CHO ratio (Fig. 10).

PRT to CHO ratio recorded higher values in this station so that we may predict plankton bloom followed by meiofaunal abundance. The elevated PRT:CHO ratio, particularly in core I, signifies heightened detritus mineralization and an associated augmentation in their protein content as a consequence of bacterial enzymatic processes, as reported in previous studies^68^. Conversely, in core II, where the PRT: CHO ratio is below 1, this is indicative of the presence of more extensively degraded organic matter^69^. Core samples from 90 to 120 cm also showed high values, which may be associated with higher productivity during the glacial-interglacial period. These samples also recorded a high PRT to CHO ratio, indicating the influence of higher productivity. The depth interval of 170–180 cm also revealed similar observation, which may be associated with higher nutrients contributed by Himalayan erosion around ≈ 0.5 Ma might reinforce higher production in the benthic community. Sample from 230 to 250 cm also showed similar observations, which may be resulted from a massive erosional event caused by the tectonic activity during ≈ 0.8 Ma from Himalaya^53^. Sample from depth interval 270–280 cm also exhibited enhancement for both LPD to CHO and PRT to CHO ratio, which could be due to higher production for meiofaunal abundance. Nevertheless, the concentration of biochemical components can be modulated during settling organic components to the sedimentary environment by diagenesis, even though it is preserved during anoxic environments. The PRT to CHO, as well as LPD to CHO ratio in core I, displayed the highest value than the other sediments from the different regions. However, these two ratios in core II are comparatively low except 10–20 cm (LPD: PRT = 0), which shows LPD to CHO ratio > 1.

Several factors can influence the processes that controls erosion, sediment accumulation, organic matter distribution, and the composition of organic molecules in the oceanic sediments. They are mainly environmental conditions, biological activity, and sedimentary processes. Erosion on land transports organic matter and minerals into rivers and eventually to the oceans. Organic matter, such as plant debris, gets mixed with soil particles during erosion. The sediments accumulates in ocean basins due to various processes like riverine input, biological activity (e.g., shell fragments), and aeolian transport. Organic matter in sediments comes from terrestrial plants, marine algae, phytoplankton, and zooplankton. The type and source of organic matter influence its composition. Over time, buried organic matter undergoes diagenesis, a series of chemical and biological changes, altering its composition and structure.

## Conclusion

The study depicted the variation of total organic carbon and biochemical parameters in the two core samples taken from the central Indian Ocean basin. Sediment core sample I was collected from the central Indian Ocean region dominated by terrigenous sediments, and Core II was collected from the eastern side of the Afanasiy-Nikitin Seamount in the Equatorial East Indian Ocean. The presence of manganese nodules and Ferromanganese crusts in the region where Core II was collected. These geological formations are notably dense and rigid, making it challenging to extract cores from deeper layers or to obtain a larger number of samples. Consequently, a restricted sample size of three samples due to a constrained sample of only 30 cm in core II.

The subsamples collected from core II also showed a higher % of sand as compared to the core I. Core I was associated with finer sediment texture with a lower amount of sand (< 1%) fraction. Higher TOC was associated with erosional events reported in the Himalayas around 0.5 Ma, ≈ 0.8 Ma and ≈ 1.7 Ma in the study region. Then the down core variation of TOC shows a decreasing trend, which could be due to the lower abundance of C4 plants in the Ganga–Brahmaputra basin^53^. Down core variation of protein up to 300 cm showed a zigzag pattern with peaks, which may indicate in situ production of organic matter during upwelling events^70,71^. The most important Pleistocene high productivity started at 1.8 Ma and lasted until − 1.2 Ma^72^, which is associated with lower protein but higher lipid content. Low protein content in this section of the core may reflect the lower bacterial activity. Sample from 270 to 280 cm also showed a higher ratio for LPD to CHO and PRT to CHO, which could be due to higher production for meiofaunal abundance. Principal component analysis showed that lipid is the major contributor to BPC in core I. The analysis also suggests that lipids and carbohydrates can be originated mainly from the oceanic environment. The amount of organic carbon and biochemical parameters may not be completely preserved in the sedimentary records due to the diagenesis by the microbial community, even though it can provide some insights into the quality of organic matter. These results can be opened up for further research with additional parameters to refine the organic source interpretations and to improve our understanding of the processes that control organic matter preservation in marine sediments during different geological periods. Changes in the depositional characteristics along a longitude in the central Indian Ocean can also be inferred from the study.

## Data Availability

Data will be made available on request. For the datasets used and/or analysed during the current study will be available from the corresponding author (mmullungal@qu.edu.qa) on reasonable request.

## References

[1] Lessin G (2018). Modelling marine sediment biogeochemistry: Current knowledge gaps, challenges, and some methodological advice for advancement. Front. Mar. Sci..

[2] Chen M, Hur J (2015). Pre-treatments, characteristics, and biogeochemical dynamics of dissolved organic matter in sediments: A review. Water Res..

[3] Sarker S, Masud-Ul-Alam M, Hossain MS, Rahman Chowdhury S, Sharifuzzaman SM (2021). A review of bioturbation and sediment organic geochemistry in mangroves. Geol. J..

[4] Graf G (1992). Benthic-pelagic coupling: A benthic view. Oceanogr. Mar. Biol. Ann. Rev..

[5] Hartnett HE, Keil RG, Hedges JI, Devol AH (1998). Influence of oxygen exposure time on organic carbon preservation in continental margin sediments. Nature.

[6] Graf G, Schulz R, Peinert R, Meyer-Reil LA (1983). Benthic response to sedimentation events during autumn to spring at a shallow-water station in the Western Kiel Bight. Mar. Biol..

[7] Rodil IF, Lastra M, López J (2007). Macroinfauna community structure and biochemical composition of sedimentary organic matter along a gradient of wave exposure in sandy beaches (NW Spain). Hydrobiologia.

[8] Viollier E, Rabouille C, Apitz SE, Breuer E, Chaillou G, Dedieu K (2003). Benthic biogeochemistry: State of the art technologies and guidelines for the future of in situ survey. J. Exp. Mar. Biol. Ecol..

[9] Dickens AF (2004). Sources, Cycling and Preservation of Black Carbon in Sediments from the Washington Margin.

[10] Fabiano M, Danovaro R, Fraschetti S (1995). A three-year time series of elemental and biochemical composition of organic matter in subtidal sandy sediments of the Ligurian Sea (northwestern Mediterranean). Cont. Shelf Res..

[11] Dell'Anno A, Mei ML, Pusceddu A, Danovaro R (2002). Assessing the trophic state and eutrophication of coastal marine systems: A new approach based on the biochemical composition of sediment organic matter. Mar. Pollut. Bull..

[12] Sardessai S, Shetye S, Maya MV, Mangala KR, Prasanna Kumar S (2010). Nutrient characteristics of the water masses and their seasonal variability in the eastern equatorial Indian Ocean. Mar. Environ. Res..

[13] Clemens SC, Murray DW, Prell WL (1996). Nonstationary phase of the Plio-Pleistocene Asian monsoon. Science.

[14] Fontugne MR, Duplessy JC (1986). Variations of the monsoon regime during the upper Quaternary: Evidence from carbon isotopic record of organic matter in North Indian Ocean sediment cores. Palaeogeogr. Palaeoclimatol. Palaeoecol..

[15] Mullungal MN, Thalayappil S, Peediyakkathodi S, Salas PM, Sujatha CH, Kumar CSR (2022). Vertical distribution of phosphorous fractions and bioavailability of the nutrient in the southern Indian Ocean. Int. J. Environ. Res..

[16] Rajani RP, Banakar VK, Parthiban G, Mudholkar AV, Chodankar AR (2005). Compositional variation and genesis of ferromanganese crusts of the Afanasiy—Nikitin Seamount, Equatorial Indian Ocean. J. Earth Syst. Sci..

[17] Jauhari P, Iyer SD (2008). A comprehensive view of manganese nodules and volcanics of the Central Indian Ocean Basin. Mar. Georesour. Geotechnol..

[18] Krumbein WC, Pettijohn FJ (1938). Manual of Sedimentary Petrography.

[19] Bouillon S, Moens T (2004). Resource utilization patterns of epifauna from mangrove forests with contrasting inputs of local versus imported organic matter. Mar. Ecol. Prog. Ser..

[20] Radojevic M, Bashkin V, Bashkin VN (1999). Practical Environmental Analysis.

[21] Dubois M, Gilles KA, Hamilton JK, Rebers PT, Smith F (1956). Colorimetric method for determination of sugars and related substances. Anal. Chem..

[22] Lowry OH, Rosebrough NJ, Farr AL (1951). Protein measurement with the Folin phenol reagent. J. Biol..

[23] Rice DL (1982). The detritus nitrogen problem: New observations and perspectives from organic geochemistry. Mar. Ecol. Prog. Ser..

[24] Barnes H, Blackstock J (1973). Estimation of lipids in marine animals and tissues: detailed investigation of the sulphophosphovanilun method for ‘total’lipids. J. Exp. Mar. Biol. Ecol..

[25] APHA (1995). Standard Methods for the Examination of Water and Wastewater.

[26] Nair RR, Ittekkot V, Manganini SJ, Ramaswamy V, Haake B, Degens ET (1989). Increased particle flux to the deep ocean related to monsoons. Nature.

[27] Fabiano M, Danovaro R (1994). Composition of organic matter in sediments facing a river estuary (Tyrrhenian Sea): Relationships with bacteria and microphytobenthic biomass. Hydrobiologia.

[28] Fichez R (1991). Composition and fate of organic-matter in submarine cave sediments-implications for the biogeochemical cycle of organic-carbon. Oceanolo Acta.

[29] Stahr HM, Hyde W, Sigler L (1982). Oxidizing acid baths—Without chromate hazards. Anal. Chem..

[30] Riley JP, Chester R (1976). Chemical Oceanography, v. 5 and 6.

[31] Rakesh S, Sinha AK, Mukhopadhyay P (2020). Vertical distribution of TOC, TN and other important soil attributes and their relationship in Alfisol and Entisol of West Bengal. Int. J. Environ. Clim. Change.

[32] Sabadini-Santos E, Senez TM, Silva TS, Moreira MR, Mendonça-Filho JG, Santelli RE, Crapez MA (2014). Organic matter and pyritization relationship in recent sediments from a tropical and eutrophic bay. Mar. Pollut. Bull..

[33] Sudheesh V, Movitha M, Hatha AM, Renjith KR, Resmi P, Nair RM (2017). Effects of seasonal anoxia on the distribution of phosphorus fractions in the surface sediments of southeastern Arabian Sea shelf. Cont. Shelf Res..

[34] Nath BN, Rao VP, Becker KP (1989). Geochemical evidence of terrigenous influence in deep-sea sediments up to 8 S in the Central Indian Basin. Mar. Geol..

[35] Mudholkar, A. V., Pattan, J. N., & Parthiban, G. Geochemistry of deep-sea sediment cores from the Central Indian Ocean Basin (1993).

[36] France-Lanord C, Derry LA (1997). Organic carbon burial forcing of the carbon cycle from Himalayan erosion. Nature.

[37] Cochran, J. R. Himalayan uplift, sea level, and the record of Bengal Fan sedimentation at the ODP leg 116 sites. In *Proceedings of the Ocean Drilling, Scientific Results* (eds. Cochran, J. R. et al.), vol. 116, 397–414 (1990).

[38] Fierens R, Toucanne S, Droz L, Jouet G, Raisson F, Jorissen (2020). Quaternary sediment dispersal in the Zambezi turbidite system (SW Indian Ocean). Mar. Geol..

[39] Jiwarungrueangkul T, Jirapinyakul A, Sompongchaiyakul P, Zhao S, Rattanakom R (2022). Response of sediment grain size to sea-level rise during the middle Holocene on the west coast of the Gulf of Thailand. Arab. J. Geosci..

[40] Danovaro R, Fabiano M, Della Croce N (1993). Labile organic matter and microbial biomasses in deep-sea sediments (Eastern Mediterranean Sea). Deep Sea Res. Part I Oceanogr. Res. Pap.

[41] Tselepides A, Polychronaki T, Marrale D, Akoumianaki I, Dell'Anno A, Pusceddu A, Danovaro R (2000). Organic matter composition of the continental shelf and bathyal sediments of the Cretan Sea (NE Mediterranean). Prog. Oceanogr..

[42] Sarnthein M, Winn K, Zahn R (1987). Paleoproductivity of oceanic upwelling and the effect on atmospheric C0 2 and climatic change during deglaciation times. Abrupt. Clim. Change.

[43] Zhao M, Mercer JL, Eglinton G, Higginson MJ, Huang CY (2006). Comparative molecular biomarker assessment of phytoplankton paleoproductivity for the last 160 kyr off Cap Blanc, NW Africa. Org. Geochem..

[44] Alagarsamy R (2003). Organic matter composition in sediments of the Oman margin. Chem. Ecol..

[45] Arndt S, Jørgensen BB, LaRowe DE, Middelburg JJ, Pancost RD, Regnier P (2013). Quantifying the degradation of organic matter in marine sediments: A review and synthesis. Earth Sci. Rev..

[46] Salas PM, Sujatha CH, Ratheesh Kumar CS (2015). Fate and source distribution of organic constituents in a river-dominated tropical estuary. J. Earth Syst. Sci..

[47] Saraswat R, Nigam R, Barreto L (2005). Palaeoceanographic implications of abundance and mean proloculus diameter of benthic foraminiferal species Epistominella exigua in sub-surface sediments from distal Bay of Bengal fan. J. Earth Syst. Sci..

[48] Punyu VR, Banakar VK, Garg A (2014). Equatorial Indian Ocean productivity during the last 33 kyr and possible linkage to Westerly Jet variability. Mar. Geol..

[49] Tiwari M, Ramesh R, Somayajulu BLK, Jull AJT, Burr GS (2006). Paleomonsoon precipitation deduced from a sediment core from the equatorial Indian Ocean. Geo-Mar. Lett..

[50] Rao VP, Kessarkar PM, Thamban M, Patil SK (2010). Paleoclimatic and diagenetic history of the late quaternary sediments in a core from the Southeastern Arabian Sea: Geochemical and magnetic signals. J. Oceanogr..

[51] Nath BN, Gupta SM, Mislankar PG, Rao BR, Parthiban G, Roelandts I, Patil SK (2005). Evidence of Himalayan erosional event at ∼ 0.5 Ma from a sediment core from the equatorial Indian Ocean in the vicinity of ODP Leg 116 sites. Deep Sea Res. II Top. Stud. Oceanogr..

[52] Valdiya KS (2002). Emergence and evolution of Himalaya: Reconstructing history in the light of recent studies. Prog. Phys. Geogr..

[53] France-Lanord C, Derry LA (1994). δ13C of organic carbon in the Bengal Fan: Source evolution and transport of C3 and C4 plant carbon to marine sediments. Geochim. Cosmochim. Acta.

[54] Valdiya KS (1999). Rising Himalaya: Advent and intensification. Curr. Sci..

[55] Sane E, Ingrassia M, Martorelli E, Chiocci FL (2020). Amino acids in surface sediments of the Zannone Island shelf (Western Mediterranean Sea): Possible bioindicators of submarine hydrothermal activity. Org. Geochem..

[56] Manju MN, Resmi P, Kumar CSR, Gireeshkumar TR, Chandramohanakumar N, Joseph MM (2016). Biochemical and stable carbon isotope records of mangrove derived organic matter in the sediment cores. Environ. Earth Sci..

[57] Baldi F, Marchetto D, Pini F, Fani R, Michaud L, Giudice AL, Berto D, Giani M (2010). Biochemical and microbial features of shallow marine sediments along the Terra Nova Bay (Ross Sea, Antartica). Cont. Shelf Res..

[58] Moers MEC, Jones DM, Eakin PA, Fallick AE, Griffiths H, Larter SR (1993). Carbohydrate diagenesis in hypersaline environments: Application of GC-IRMS to the stable isotope analysis of derivatized saccharides from surficial and buried sediments. Org. Geochem..

[59] Patriquin DG (1972). The origin of nitrogen and phosphorus for growth of the marine angiosperm Thalassia testudinum. Mar. Biol..

[60] Cividanes S, Incera M, López J (2002). Temporal variability in the biochemical composition of sedimentary organic matter in an intertidal flat of the Galician coast (NW Spain). Oceanolo Acta.

[61] Lee S, Fuhrman JA (1987). Relationships between biovolume and biomass of naturally derived marine bacterioplankton. Appl. Environ. Microbiol..

[62] Ogier S, Disnar JR, Albéric P, Bourdier G (2001). Neutral carbohydrate geochemistry of particulate material (trap and core sediments) in an eutrophic lake (Aydat, France). Org. Geochem..

[63] Pusceddu A, Sarà G, Mazzola A, Fabiano M (1997). Relationships between suspended and sediment organic matter in a semi-enclosed marine system: The Stagnone di Marsala sound (Western Sicily). Wate Air Soil Pollut..

[64] Fabiano M, Pusceddu A (1998). Total and hydrolizable particulate organic matter (carbohydrates, proteins and lipids) at a coastal station in Terra Nova Bay (Ross Sea, Antarctica). Polar Biol..

[65] Grémare A, Medernach L, DeBovee F, Amouroux JM, Vétion G, Albert P (2002). Relationships between sedimentary organics and benthic meiofauna on the continental shelf and the upper slope of the Gulf of Lions (NW Mediterranean). Mar. Eco. Prog. Ser..

[66] Gremare A, Amouroux JM, Charles F, Dinet A, Riaux-Gobin C, Baudart J (1997). Temporal changes in the biochemical composition and nutritional value of the particulate organic matter available to surface deposit-feeders: A two year study. Mar. Ecol. Prog. Ser..

[67] Cartes JE, Grémare A, Maynou F, Villora-Moreno S, Dinet A (2002). Bathymetric changes in the distributions of particulate organic matter and associated fauna along a deep-sea transect down the catalan sea slope (Northwestern Mediterranean). Prog. Oceanogr..

[68] Venturini N, Pita AL, Brugnoli E, García-Rodríguez F, Burone L, Kandratavicius N, Muniz P (2012). Benthic trophic status of sediments in a metropolitan area (Rio de la Plata estuary): Linkages with natural and human pressures. Estuarine Coast. Shelf Sci..

[69] Danovaro R, Fabiano M, Della Croce N (1993). Labile organic matter and microbial biomasses in deep-sea sediments (Eastern Mediterranean Sea). Deep Sea Res..

[70] Stuhldreier I, Sánchez-Noguera C, Rixen T, Cortés J, Morales A, Wild C (2015). Effects of seasonal upwelling on inorganic and organic matter dynamics in the water column of eastern Pacific coral reefs. PloS One.

[71] Bode A, Álvarez M, Ruíz-Villarreal M (2019). Changes in phytoplankton production and upwelling intensity off A Coruña (NW Spain) for the last 28 years. Ocean Dyn..

[72] Gupta AK, Thomas E (1999). Latest miocene-pleistocene productivity and deep-sea ventilation in the northwestern Indian Ocean (deep sea drilling project site 219). Paleoceanography.

